# Surgical results in acute type A aortic dissection with preoperative cardiopulmonary resuscitation: Survival and neurological outcome

**DOI:** 10.1371/journal.pone.0237989

**Published:** 2020-08-24

**Authors:** Chun-Yu Lin, Chi-Nan Tseng, Cheng-Hui Lu, Tao-Hsin Tung, Feng-Chun Tsai, Meng-Yu Wu

**Affiliations:** 1 Department of Medicine, College of Medicine, Chang-Gung University, Taoyuan, Taiwan; 2 Department of Cardiothoracic and Vascular Surgery, Chang-Gung Memorial Hospital, Linkou Medical Center, Taoyuan, Taiwan; 3 Department of Cardiothoracic and Vascular Surgery, Chang-Gung Memorial Hospital, Tucheng branch, New Taipei, Taiwan; 4 Department of Cardiology, Chang-Gung Memorial Hospital, Linkou Medical Center, Taoyuan, Taiwan; 5 Department of Medical Research and Education, Cheng-Hsin General Hospital, Taipei, Taiwan; IRCCS Policlinico S.Donato, ITALY

## Abstract

**Background:**

Acute type A aortic dissection (ATAAD) is a life-threatening disease that requires emergent surgical intervention. This retrospective study aimed to clarify the individual characteristics, short-term and mid-term outcomes, and prognostic factors of patients who underwent surgical repair of ATAAD with preoperative cardiopulmonary resuscitation (CPR).

**Methods:**

Between January 2007 and January 2020, 656 consecutive patients underwent ATAAD repair at our institution; 22 (3.4%) of these patients underwent CPR prior to surgery. Patients who underwent preoperative CPR were classified as the survivor group (n = 9) and non-survivor group (n = 13), according to whether they survived to hospital discharge. Clinical features, surgical information, and postoperative complications were analyzed and compared. Three-year cumulative survival rates and cerebral performance categories (CPC) scores are presented.

**Results:**

In patients undergoing CPR prior to ATAAD surgery, the in-hospital mortality rate was 59.1%. A total of 72.7% of patients underwent concomitant surgical resuscitation procedures during CPR such as emergent subxiphoid pericardiotomy and/or emergent cardiopulmonary bypass. The survivor group had a higher rate of return of spontaneous heartbeat (ROSB) compared to the non-survivor group (100% versus 53.8%; *P* = 0.017). The 3-year cumulative survival rates were 35.1% (95% confidence interval [CI], 27.6%–42.6%) and 85.7% (95% CI, 81.9%–88.8%) for overall patients and for survivors, respectively. As for the neurological outcome, 77.8% (7/9) of patients had full cerebral performance (CPC-1) at the 3-month follow-up examination after discharge.

**Conclusions:**

Patients with ATAAD undergoing preoperative CPR, especially those without ROSB after CPR, are at high risk for in-hospital mortality. However, the short-term and mid-term outcomes, including the cerebral performance after discharge and 3-year survival rate, are promising for patients who survived to discharge.

## Introduction

It is widely accepted that acute type A aortic dissection (ATAAD) is a life-threatening condition associated with high morbidity and mortality rates. ATAAD is also a challenge for cardiothoracic surgeons because of the complex anatomy of the aorta and possibility of preoperative complications, including hemodynamic collapse requiring cardiopulmonary resuscitation (CPR). Previous studies reported that the incidence of preoperative CPR ranges from 3.8% to 6.3% [1–3]. Among these patients, the in-hospital mortality rates ranged from 50% to 95% [1,4,5], which was approximately five-times to six-times higher than that of the general ATAAD population [6,7]. However, even though they comprise an extremely high-risk subgroup, the clinical characteristics and surgical results of patients with ATAAD undergoing preoperative CPR may be under-reported. This study was based on a retrospective analysis of the experiences of an aortic surgery center and aimed to present the individual characteristics, short-term and mid-term outcomes, and prognostic factors of patients who underwent CPR before surgical repair for ATAAD.

## Material and methods

### Patient enrollment

**Fig 1:**
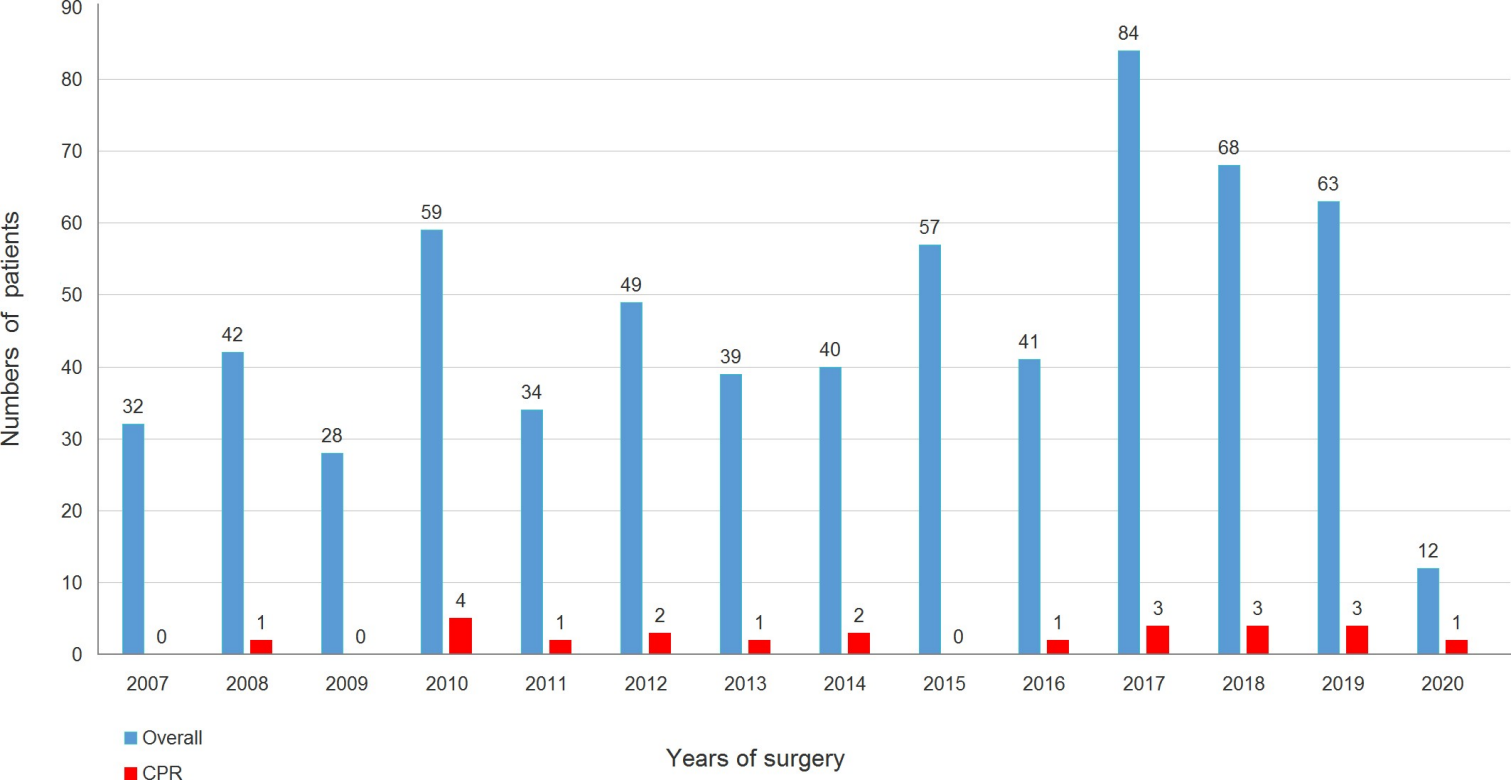
Distribution of ATAAD patients during the study period. ATAAD, acute type A aortic dissection.

The study protocol was approved by the Chang-Gung Medical Foundation Institutional Ethics Committee (No.202000397B0). The requirement for informed consent was waived due to the retrospective design of the study. Overall, 656 consecutive adult patients underwent emergent ATAAD repair at our institution between January 2007 and January 2020. All patients were diagnosed via helical computed tomography in the emergency department (ED), and the extent of aortic dissection, presence of organ malperfusion, and hemopericardium were analyzed. A total of 22 (3.4%) patients who underwent CPR before surgery were included. The overall number of patients per year who underwent ATAAD repair and the number of patients per year who underwent CPR before surgery during the study period are illustrated in Fig 1. The details of each patient’s individual characteristics regarding CPR, type of aortic repair procedure, and final outcome are summarized in Table 1. The included patients were dichotomized into two groups, the survivor group (n = 9) and non-survivor (n = 13) group, according to whether they survived to hospital discharge.

**Table 1:** Characteristics of CPR, type of aortic repair procedures, and outcomes.

No.	Causes of hemodynamic instability	CPR location	CPR duration (min)	Surgical resuscitation procedures	Aortic repair procedures	Outcomes
1	Tamponade	OR	Uncertain	E-SXP	AsAo replacement	Survived
2	Tamponade	OR	30	E-SXP + E-CPB	AsAo replacement	Survived
3	Tamponade + AMI	ED	10	E-SXP + E-CPB	AsAo replacement	Dead
4	AMI	OR	30	E-CPB	Aortic root replacement	Dead
5	Tamponade	ED	25	E-SXP	AsAo replacement	Dead
6	Uncertain	OR	30	—	AsAo replacement	Dead
7	Tamponade	OR	15	E-SXP + E-CPB	AsAo replacement	Dead
8	Tamponade + aortic rupture	OR	22	E-CPB	AsAo replacement	Survived
9	Tamponade + aortic rupture	OR	Uncertain	E-SXP + E-CPB	AsAo + partial arch replacement	Survived
10	Tamponade	ED	20	—	AsAo replacement	Survived
11	Tamponade	OR	31	E-SXP	AsAo replacement	Survived
12	Tamponade	OR	5	E-SXP	AsAo replacement	Survived
13	Uncertain	OR	Uncertain	—	AsAo replacement	Dead
14	Tamponade	OR	Uncertain	—	AsAo replacement	Survived
15	Tamponade	ED	1	E-SXP	AsAo replacement	Dead
16	Severe AR with heart failure	ED	6	—	AsAo replacement	Dead
17	Tamponade	OR	3	E-SXP	AsAo replacement	Dead
18	Tamponade + aortic rupture	OR	60	E-CPB	AsAo replacement	Dead
19	AMI	OR	3	—	AsAo replacement	Survived
20	Tamponade	ED	15	E-CPB	AsAo replacement	Dead
21	Tamponade	OR	Uncertain	E-SXP	AsAo replacement	Dead
22	Severe AR with heart failure	OR	20	E-CPB	Aortic root replacement	Dead

AMI, acute myocardial infarction; AR, aortic regurgitation; AsAo, ascending aorta; E-CPB, emergent cardiopulmonary bypass; ED, emergency department; E-SXP, emergent subxiphoid pericardiotomy; OR, operating room.

### Preoperative management and CPR

Based on a strict institutional protocol, when a diagnosis of ATAAD was confirmed, patients were emergently transferred to the operating room to avoid delays in aortic repair surgery. If patients presented with hypertension or tachycardia before surgery, then their hemodynamics were stabilized with intravenous beta-blockers to maintain the systolic blood pressure (SBP) at <120 mmHg and heart rate at 60–70 bpm according to the 2010 American College of Cardiology/American Heart Association guidelines for thoracic aortic disease [8]. When patients presented with shock, medical resuscitation was applied first, including intravenous fluid supplementation and inotropic infusion. For refractory hemodynamic instability or cardiac arrest, CPR was performed according to the established guidelines of advanced cardiovascular life support (ACLS) developed by American Heart Association during the study period [9–11]. All included patients underwent in-hospital CPR which was performed by physicians with ACLS certification. Based on a previous study performed at our institution, surgical resuscitation procedures, including emergent subxiphoid pericardiotomy and/or emergent cardiopulmonary bypass, were implemented if necessary according to the individual patient’s circumstance [12]. In general, emergent subxiphoid pericardiotomy was performed if cardiac tamponade was confirmed based on preoperative imaging studies and on-site echocardiography; emergent cardiopulmonary bypass was performed if aortic rupture was suspected or if patients presented with refractory cardiac arrest without return of spontaneous circulation after CPR and medical resuscitation. All management strategies were performed on an emergency basis.

### ATAAD repair procedures

The general principles of ATAAD repair procedures have been detailed in previous studies reported by our institution [12,13]. For patients who presented with an unstable hemodynamic status before surgery, especially those who required CPR, the femoral artery was considered a preferable choice for cannulation access to minimize the time required to establish cardiopulmonary bypass (CPB). We routinely used retrograde cerebral perfusion (RCP) with deep hypothermia for patients who underwent solitary femoral arterial cannulation. Otherwise, an additional right axillary arterial cannulation was implemented and connected with the femoral arterial cannula with a Y-shape circuit if antegrade cerebral perfusion (ACP) was applied. In general, a conservative strategy involving aortic repair procedures was preferable for these high-risk patients. In most cases, isolated ascending aortic replacement with a Dacron prosthetic graft was performed, regardless of the location of the entry tear. The proximal anastomosis was usually performed first, followed by open distal anastomosis under circulatory arrest with deep hypothermia. Aortic root replacement was performed with a composite Valsalva graft if the intimal tear that extended to the aortic root was considered difficult to repair. After undergoing surgical repair for ATAAD, all patients were transferred to a specialized cardiovascular intensive care unit (ICU) for further treatment and observation.

### Data collection and statistical analyses

**Fig 2:**
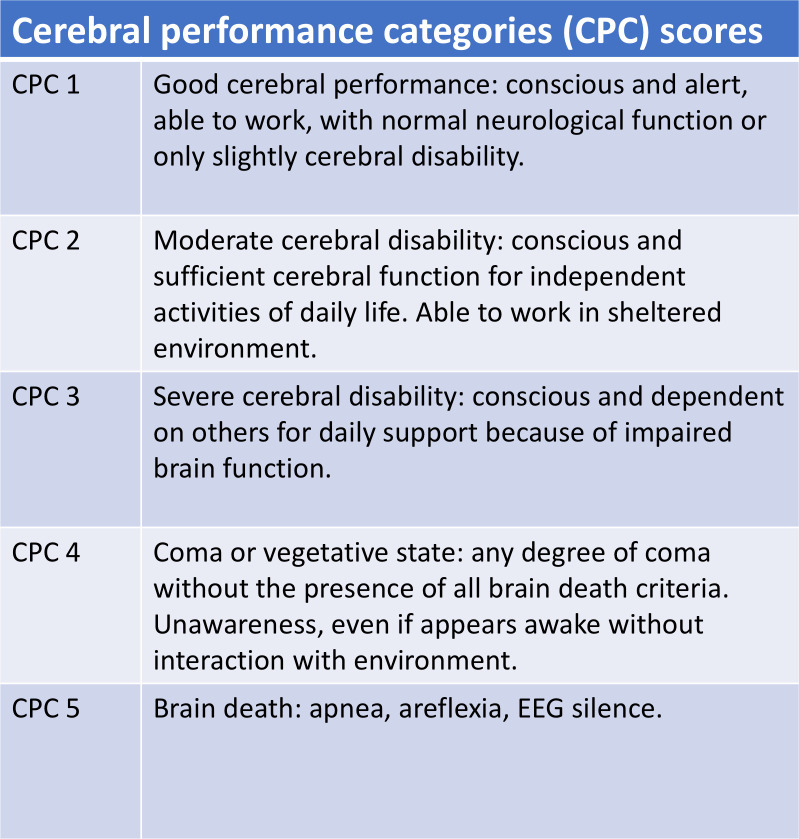
Cerebral performance categories (CPC) scores.

All data collection was performed through the electronic medical records, which were accessed from January 2007 to April 2020. Statistical analyses were performed using SPSS for Windows (version 22.0; IBM Corp., Armonk, NY). Clinical features, CPR-related profiles, surgical information, and postoperative complications of the survivor and non-survivor groups were analyzed and compared. Data are presented as medians and interquartile ranges for numerical variables and as numbers/percentages for categorical variables. For intergroup comparisons, we used the Mann–Whitney U-test for numerical variables and chi-square or the Fisher’s exact test for categorical variables. The Kaplan–Meier method was used to estimate the 3-year cumulative survival rates for all included patients and for survivors. In the Kaplan–Meier survival analysis, each patient was characterized by the time of follow-up and the status at the end of follow-up (live/dead). Patients died within a 3-year follow-up was defined as event occurrence; patients lived at the end of follow-up less than 3 years was defined as censored. Two patients were censored in the survival analysis (case 1, 3-month follow-up; case 2, 6-month follow-up). Total probability of survival was calculated by multiplying the probabilities of each time interval. To evaluate the neurological outcomes among surviving patients, the cerebral performance categories (CPC) scores were analyzed individually at discharge and 3 months after discharge [14]. The CPC score ranges from 1 to 5 with 1 representing good cerebral performance and 5 representing brain death. The detailed classification is illustrated in Fig 2. A propensity score-matched analysis was conducted to compare patients with and without preoperative CPR. The propensity score for each patient was calculated by binary logistic regression including all the variables listed in Tables 2 and 3. Patients with and without CPR were matched in a 1:1 ratio, resulting in 22 matched pairs in whom the postoperative outcomes were assessed and compared. For all analyses, statistical significance was set at *P* < 0.05.

**Table 2:** Preoperative characteristics according to the patient group.

Parameters	Overall	Survivors	Non-survivors	*P* value
	n = 22	n = 9	n = 13	
Clinical demographics				
Sex (female, n, %)	8, 36.4	3, 33.3	5, 38.5	0.806
Age (year)	63.1 (54.9–76.5)	63.6 (54.2–72.3)	62.6 (47.8–86.0)	0.764
Body mass index (kg/m^2^)	25.1 (23.0–27.6)	24.2 (23.0–27.4)	26.0 (22.9–27.8)	0.867
Hypertension (n, %)	14, 63.6	5, 55.6	9, 69.2	0.512
Diabetes mellitus (n, %)	3, 13.6	1, 11.1	2, 15.4	0.774
Creatinine (mg/dL)	1.6 (1.0–2.0)	1.6 (1.0–2.0)	1.6 (1.0–1.9)	0.920
eGFR (mL/min/1.73 m_2_)	43.9 (34.8–72.2)	45.1 (36.1–69.4)	42.6 (33.8–79.6)	0.920
Preoperative condition and CPR-related profiles				
SBP (mmHg)	50.0 (45.0–57.0)	50.0 (48.0–56.0)	50.0 (45.0–59.5)	0.761
SBP <60 mmHg (n, %)	18, 81.8	8, 88.9	10, 76.9	0.474
Time from ED to OR (hr)	4.3 (2.5–6.5)	4.7 (2.7–7.3)	3.9 (2.2–5.8)	0.615
CPR at ED (n, %)	6, 27.3	1, 11.1	5, 38.5	0.157
CPR duration (min)	20.0 (5.5–30.0)	21.0 (4.5–30.3)	15.0 (6.0–30.0)	0.762
Surgical resuscitation procedures (n, %)	16, 72.7	6, 66.7	10, 76.9	0.595
ROSB (n, %)	16, 72.7	9, 100	7, 53.8	0.017
Clinical presentation				
Intractable pain (n, %)	13, 59.1	5, 55.6	8, 61.5	0.779
Aortic regurgitation > moderate (n, %)	7, 31.8	2, 22.2	5, 38.5	0.421
Hemopericardium (n, %)	17, 77.3	8, 88.9	9, 69.2	0.279
Cardiac tamponade (n, %)	16, 72.7	8, 88.9	8, 61.5	0.157
Acute myocardial infarction (n, %)	3, 13.6	1, 11.1	2, 15.4	0.774
Malperfusion^a^ (n, %)	10, 45.5	4, 44.4	6, 46.2	0.937
DeBakey type II (n, %)	7, 31.8	3, 33.3	4, 30.8	0.899

^a^Stroke in 5, limb ischemia in 2, and coronary artery occlusion in 3 patients.

CPR, cardiopulmonary resuscitation; eGFR, estimated glomerular filtration rate; ED, emergency department; OR, operating room; ROSB, return of spontaneous heartbeat; SBP, systolic blood pressure.

**Table 3:** Surgical information according to patient group.

Parameters	Overall	Survivors	Non-survivors	*P* value
	n = 22	n = 9	n = 13	
Femoral arterial cannulation (n, %)	21, 95.5	8, 88.9	13, 100	0.219
Axillary arterial cannulation (n, %)	8, 36.4	4, 44.4	4, 30.8	0.512
Aortic repair procedures				
Entry tear exclusion (n, %)	17, 77.3	7, 77.8	10, 76.9	0.962
Root replacement (n, %)	2, 9.1	0	2, 15.4	0.217
Isolated AsAo replacement (n, %)	19, 86.4	8, 88.9	11, 84.6	0.774
Arch replacement (n, %)	1, 4.5	1, 11.1	0	0.219
Partial arch (n, %)	1, 4.5	1, 11.1	0	0.219
Total arch (n, %)	0	0	0	0.999
Cardiopulmonary bypass time (min)	264.0 (206.5–359.8)	253.0 (184.0–334.0)	327.0 (210.0–395.5)	0.423
Aortic clamping time (min)	155.0 (130.0–209.5)	158.0 (129.0–202.0)	154.0 (125.0–247.0)	0.593
Circulatory arrest time (min)	44.5 (36.8–58.5)	39.0 (37.0–58.5)	52.0 (31.0–60.0)	0.593
ACP (n, %)	8, 36.4	4, 44.4	4, 30.8	0.512
RCP (n, %)	14, 63.6	5, 55.6	9, 69.2	0.512
Hypothermia temperature (°C)	20.0 (18.0–20.0)	20.0 (19.5–20.0)	19.0(18.0–20.0)	0.148
Delayed sternum closure^a^ (n, %)	5, 22.7	3, 33.3	2, 15.4	0.323
ECMO support (n, %)	3, 13.6	1, 11.1	2, 15.4	0.774

^a^Kerlix packing for uncontrolled coagulopathy and planned secondary exploration.

ACP, antegrade cerebral perfusion; AsAo, ascending aorta; ECMO, extracorporeal membrane oxygenation; RCP, retrograde cerebral perfusion.

## Results

### Patient demographics

As illustrated in Table 2, clinical demographics, preoperative conditions, CPR-related profiles, and clinical presentations were generally homogenous between the survivor and non-survivor groups, except for the rates of return of spontaneous heartbeat (ROSB) after CPR. The survivor group had a higher ROSB rate than the non-survivor group (100% versus 53.8%; *P* = 0.017). Overall, 36.4% of patients were female, and the median age was 63.1 years (range, 54.9–76.5 years). A total of 27.3% of patients underwent CPR in the ED, and 72.7% underwent concomitant surgical resuscitation procedures. The median CPR duration was 20.0 minutes (range, 5.5–30 minutes). Hemopericardium and cardiac tamponade were the most common clinical presentations, accounting for >70% of patients, followed by intractable chest/back pain and end-organ malperfusion.

### Surgical information

Table 3 provides detailed information regarding surgical variables. There were no intergroup differences among cannulation access, aortic repair procedures, and CPB-related profiles. Femoral artery is a commonly selected access of cannulation that was used for 95.5% of patients, and 63.6% underwent RCP during circulatory arrest. Only 36.4% of patients underwent additional axillary arterial cannulation using the ACP strategy. Isolated ascending aortic replacement is a commonly adopted aortic repair procedure that was used for >80% of patients in both groups. A total of 9.1% of patients underwent aortic root replacement, and 4.5% underwent partial aortic arch replacement. The overall entry tear exclusion rate was 77.3%. Furthermore, 13.6% of patients required intraoperative extracorporeal membrane oxygenation (ECMO) due to myocardial failure and difficulty weaning from CPB.

### Postoperative complications

As illustrated in Table 4, the in-hospital mortality rate was 59.1%. Myocardial failure was the most common cause of mortality (46.2%), followed by bleeding (23.1%), brain stem failure (15.4%), and sepsis (15.4%). High incidences of postoperative complications were observed, including acute renal failure (13.6%), re-exploration for bleeding (22.7%), brain stroke (27.3%), malperfusion-related complications (31.8%), and pneumonia (22.7%). However, there were no intergroup disparities in these complication rates. The overall median durations of ICU and hospital stays were 3.5 days (range, 0.5–17.0 days) and 13.5 days (range, 0.5–30.3 days), respectively.

**Table 4:** Postoperative mortality and morbidity according to patient group.

Parameters	Overall	Survivors	Non-survivors	*P* value
	n = 22	n = 9	n = 13	
Cause of mortality				
Bleeding (n, %)	3, 13.6	0	3, 23.1	N/A
Myocardial failure (n, %)	6, 27.3	0	6, 46.2	N/A
Brain stem failure (n, %)	2, 9.1	0	2, 15.4	N/A
Sepsis (n, %)	2, 9.1	0	2, 15.4	N/A
Renal failure (n, %)	3, 13.6	1, 11.1	2, 15.4	0.774
Transfusion at 24 hr after surgery				
RBC^a^ (units)	6.0 (5.5–12.5)	6.0 (4.0–7.0)	6.0 (6.0–17.0)	0.253
Plasma^b^ (units)	6.0 (4.0–10.5)	6.0 (4.0–8.0)	6.0 (5.0–14.0)	0.476
Platelet (units)	12.0 (12.0–24.0)	12.0 (12.0–18.0)	12.0 (12.0–24.0)	0.521
Re-exploration for bleeding (n, %)	5, 22.7	3, 33.3	2, 15.4	0.323
Atrial fibrillation (n, %)	1, 4.5	1, 11.1	0	0.219
Brain stroke (n, %)	6, 27.3	3, 33.3	3, 23.1	0.595
Infarction (n, %)	6, 27.3	3, 33.3	3, 23.1	0.595
Hemorrhage (n, %)	1, 4.5	1, 11.1	0	0.219
Delirium (n, %)	5, 22.7	2, 22.2	3, 23.1	0.962
Seizure (%n,)	2, 9.1	1, 11.1	1, 7.7	0.784
Visceral ischemia (n, %)	0	0	0	0.999
Limb ischemia (n, %)	1, 4.5	1, 11.1	0	0.219
Malperfusion-related complications^c^ (n, %)	7, 31.8	3, 33.3	4, 30.8	0.899
Pneumonia (n, %)	5, 22.7	1, 11.1	4, 30.8	0.279
ICU stay (days)	3.5 (0.5–17.0)	7.0 (3.5–12.5)	1.0 (0.5–23.5)	0.101
Hospital stay (days)	13.5 (0.5–30.3)	26.0 (13.5–33.5)	1.0 (0.5–27.0)	0.025

^a^Red blood cell transfusion including amounts of whole blood and packed red cell concentrate.

^b^Plasma transfusion including amounts of fresh-frozen plasma and cryoprecipitate.

^c^Occurrence of postoperative renal failure, brain infarction, visceral ischemia, and limb ischemia.

ICU, intensive care unit.

### Cumulative 3-year survival rate

**Fig 3:**
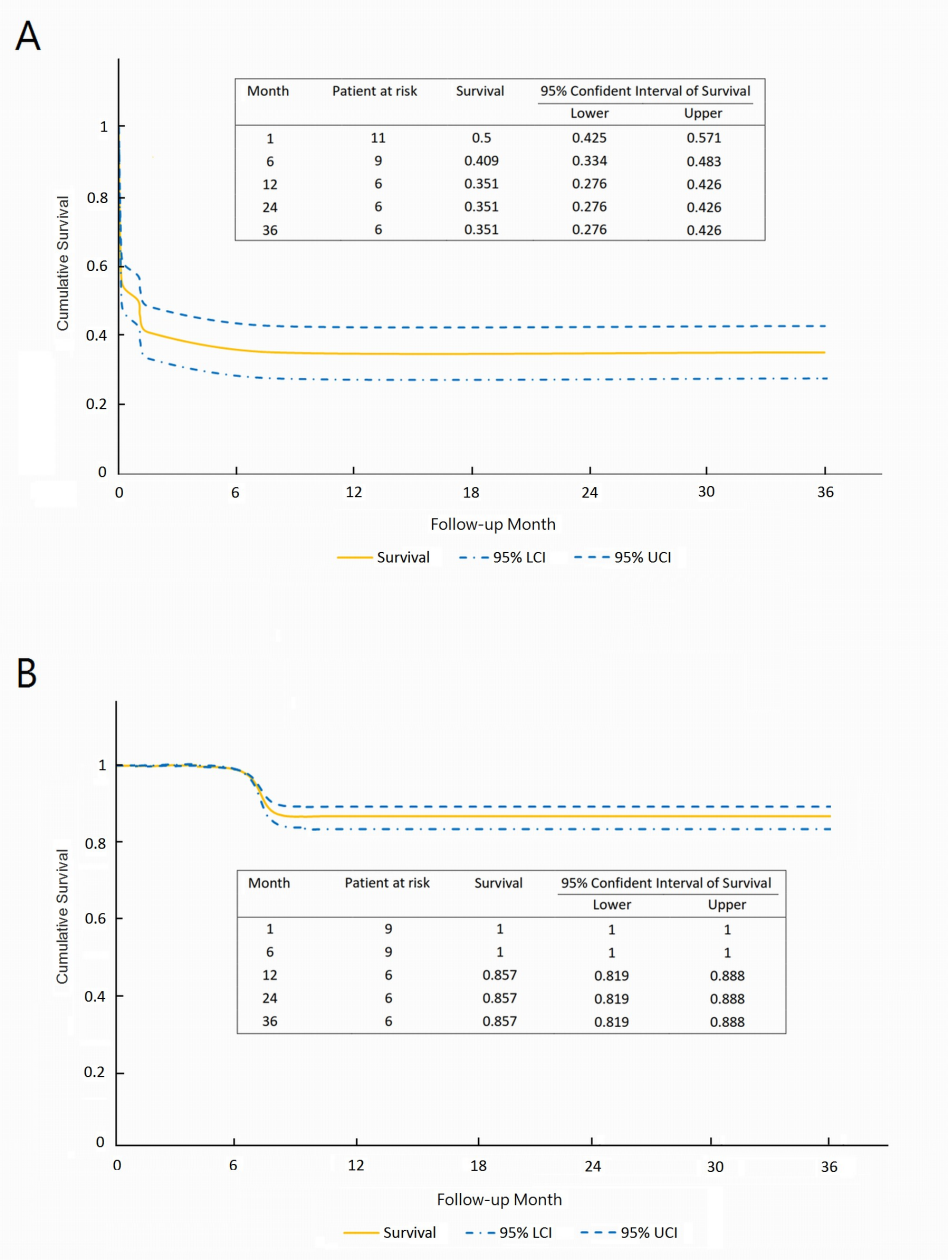
Three-year cumulative survival rates for 22 patients including in-hospital mortality (A) and for 9 patients who survived to discharge (B).

Follow-up was completed by all patients with an average of 1.4 ± 2.6 years (median, 0.2 years; range, 0.1–9.7 years). There was one case of late mortality; case 10 died due to cerebral infarction complicated by pneumonia at 8 months after discharge. As illustrated in Fig 3, the 3-year cumulative survival rates for the overall cohort and for patients who survived to discharge were 35.1% (95% confidence interval [CI], 27.6%–42.6%) (Fig 3A) and 85.7% (95% CI, 81.9%–88.8%) (Fig 3B), respectively.

### Cerebral performance

**Fig 4:**
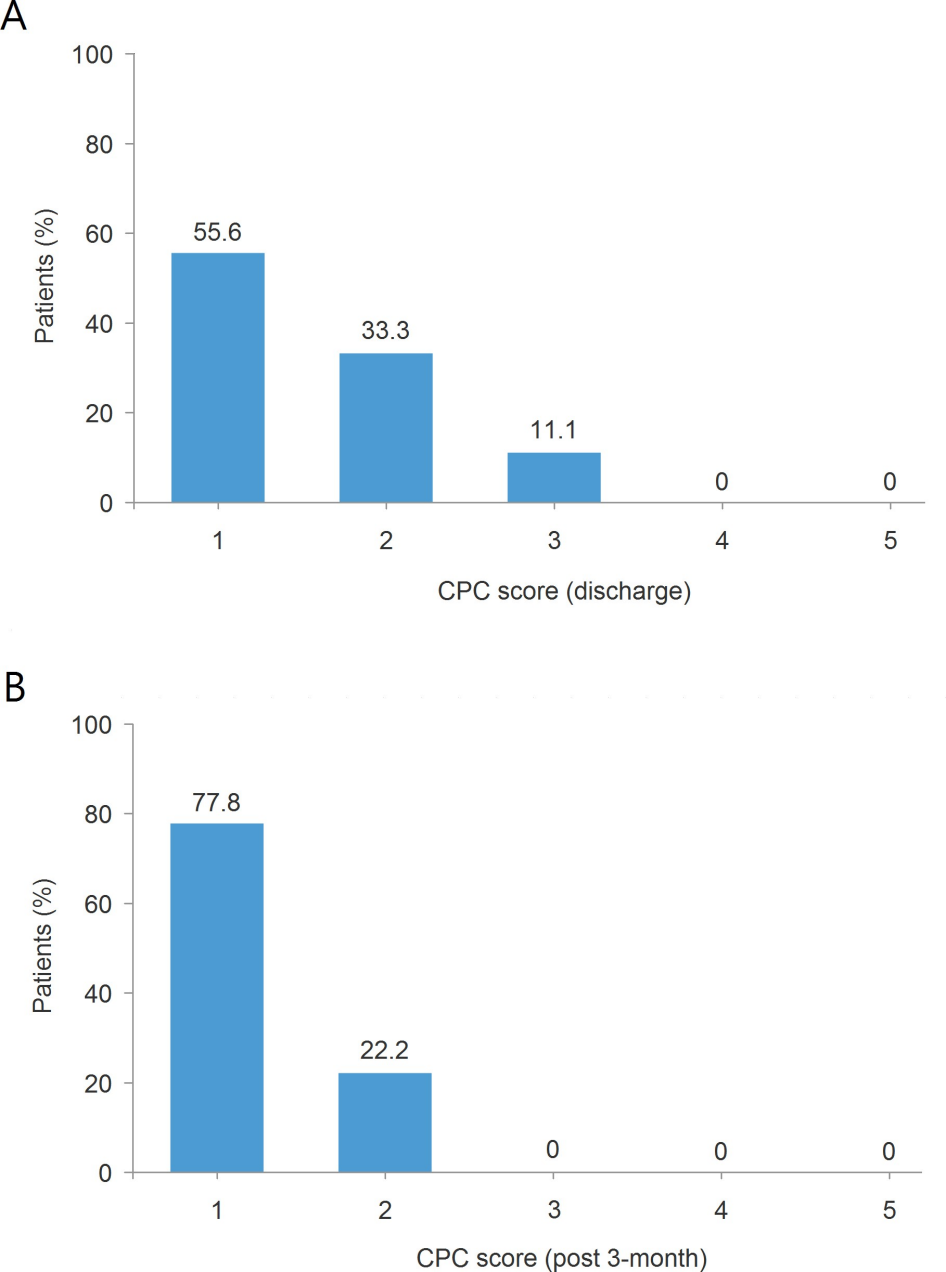
CPC score for 9 survivors at discharge (A) and at 3 months after discharge (B). CPC, cerebral performance categories.

The distribution of the CPC scores of nine survivors is illustrated in Fig 4. When patients were discharged, their CPC scores were consolidated into the following groups: CPC-1 (55.6%), CPC-2 (33.3%), and CPC-3 (11.1%) (Fig 4A). At 3 months after discharge, the prevalence of CPC-1 increased to 77.8%, and that of CPC-2 decreased to 22.2% (Fig 4B).

### Comparisons between patients with and without preoperative CPR

As illustrated in S1 Table, several preoperative and surgical variables show intergroup differences, including age, preoperative renal function, SBP, surgical resuscitation procedures, clinical presentations, access of arterial cannulation, extent of aortic repair procedures, cerebral perfusion strategy, and rate of ECMO support. S2 Table illustrates that all of the factors described above were homogenized using propensity score-matching, except the preoperative SBP (58.5 mmHg [range, 55.0–66.3 mmHg] versus 50.0 mmHg [45.0–57.0 mmHg]; *P* = 0.001), rates of axillary arterial cannulation (72.7% versus 36.4%; *P* = 0.015), and antegrade/retrograde cerebral perfusion (72.7% versus 36.4%; *P* = 0.015, 27.3% versus 63.6%; *P* = 0.015). In regard to postoperative outcomes, patients with preoperative CPR had higher rates of in-hospital mortality (18.2% versus 59.1%; *P* = 0.005) and myocardial failure as the cause of mortality (4.5% versus 27.3%; *P* = 0.039) compared to those without CPR (S3 Table).

## Discussion

In previous literatures, aortic dissection and rupture were identified as the immediate cause of death for 2.3% of patients with sudden cardiac arrest [4], and up to 18–62% of patients did not reach the hospital alive [15,16]. These risks may be more significant for patients with ATAAD due to the occurrence of cardiac tamponade, frank aortic rupture, and severe end-organ malperfusion. Approximately 3.8–6.3% of ATAAD patients presented with a critical hemodynamic status and required CPR to maintain systemic circulation until surgery [1–3]. During this study, 22 patients who underwent ATAAD repair surgeries and preoperative CPR were analyzed. This study yielded two main findings. First, all patients without ROSB did not survive after ATAAD surgery. Second, as expected, we found high in-hospital mortality and postoperative complication rates. In contrast, the short-term and mid-term outcomes for patients who survived to hospital discharge were acceptable, including the cerebral performance after discharge and 3-year survival rate. However, we believe that it should be possible to optimize the results by improving the surgical strategy and perioperative management.

### Causes of hemodynamic instability

As reported by Bossone et al., preoperative SBP <80 mmHg was an independent predictor of in-hospital mortality for patients with acute aortic dissection [17]. In the present study, approximately 80% of patients presented with SBP <60 mmHg; the average SBP was 50 mmHg before undergoing CPR. Therefore, these patients were expected to be at high risk for in-hospital mortality. Hemodynamic instability of ATAAD patients was usually associated with cardiac tamponade induced by hemopericardium, massive blood loss induced by aortic rupture, acute decompensated heart failure due to severe valvular insufficiency, and vital organ malperfusion, such as acute myocardial infarction (AMI). A total of 72.7% (16/22) of patients presented with cardiac tamponade as a cause of hemodynamic collapse, and 50% (8/16) of these patients survived. Compared to that, patients with other causes of hemodynamic instability had much lower survival rates; 33.3% (1/3) with AMI, 0% (0/2) with severe aortic regurgitation with heart failure, and 0% (0/2) with an uncertain cause of hemodynamic collapse survived, respectively. We suspect that this finding may be explained with several reasons. First, cardiac tamponade during ATAAD was usually induced by hemorrhagic leakage from the dissected ascending aorta, which accumulated in the pericardial space and compromised the venous return and limited cardiac output. Prompt and accurate management including medical resuscitation and surgical rescue procedures could efficiently reverse critical hemodynamics. During previous studies reported from this institution, a comprehensive strategy of rescuing this critical population was introduced and resulted in good outcomes [12]. Therefore, we believe that ATAAD complicated by cardiac tamponade plus CPR should be treated aggressively to achieve relatively higher survival. Second, only one patient (case 19) who underwent CPR resulted from AMI survived in this cohort. Furthermore, 66.7% (2/3 patients; cases 3 and 4) required intraoperative ECMO implementation due to myocardial failure. A previous study based on the International Registry of Acute Aortic Dissection database indicated that coronary malperfusion complicates 9–15% of ATAAD cases and is associated with an increased risk of operative mortality [18]. Regarding these patients, the occurrence of AMI was highly suspected to be associated with coronary dissection or occlusion induced by ATAAD. Therefore, even with continuous cardiac massage or emergent cardiopulmonary bypass, coronary malperfusion remains unsolved and myocardial ischemia persists. Cardioplegic-induced cardiac arrest is mandatory for aortic repair surgery. However, this essential management may worsen myocardial injury during the acute stage. Additionally, ATAAD repair surgery is a complex procedure with an extended process. Even with successful revascularization achieved by surgery, irreversible myocardial damage may develop during long-term ischemia. Emergency percutaneous coronary intervention has been reported as a life-saving modality for treating coronary malperfusion complicated by ATAAD [19,20]. Therefore, during this critical scenario, it may be helpful to seriously consider emergency percutaneous coronary intervention as a bridge treatment to surgery for anatomically suitable patients. Finally, two patients (cases 6 and 13) underwent CPR for uncertain causes. No hemopericardium, aortic rupture, signs of myocardial infarction, or valvular insufficiency were observed in these patients. However, both of them died due to uncontrolled bleeding 1 day after surgery. Even with aggressive re-exploration, it was still difficult to achieve sufficient hemostasis because of the fragile aortic tissue and severe coagulopathy found in these patients. In the context of this subgroup, surgical treatment remains challenging, and delicate aortic repair may be substantial for improving the outcomes.

### ROSB after CPR

In the present study, all patients in the survivor group and 53.8% patients in the non-survivor group had ROSB before proceeding ATAAD repair surgery. In other words, no patients survived if ROSB was absent after undergoing CPR. Even with aggressive surgical rescue procedures, including emergent cardiopulmonary bypass, which provides a similar mechanism of hemodynamic support as extracorporeal cardiopulmonary resuscitation, six patients (cases 3, 4, 7, 18, 20, 22) did not have ROSB, and all died after surgery, including 3 with myocardial failure, 2 with brain stem failure, and 1 with sepsis plus multiple organ failure. This disastrous outcome may be explained with several reasons. First, external cardiac compression generates only 25% to 33% of normal cardiac output [21]. In this low-flow state, tissue hypoxia will persist until effective spontaneous perfusion is restored or extracorporeal circulation is initiated. However, an irreversible damage to the vital organs, especially the heart and brain may have occurred. All the 6 patients did not have ROSB even though emergent cardiopulmonary bypass was implemented, and most of these patients presented with extensively nonviable myocardial tissue and cardiac asystole after sternotomy. Furthermore, the cardiac contractility of these patients did not show any significant improvement after aortic repair surgeries were completed. Three of these patients died in OR, and the other three died in 48 hrs. Second, the possible ways to treat such patients with irreversible myocardial dysfunction include heart transplantation and implantation of ventricular device. However, these modalities were not indicated on patients with severe cerebral damage according to current guidelines [22,23], and can not be considered as a preferable treatment option before confirming these patients’ cerebral function were intact.

We believe this is an important finding which may indicate that patients with refractory cardiac arrest without ROSB may be eligible for conservative surgical treatment because extremely low survival is expected. As reported by Lin and Lee et al., the absence of ROSB was associated with inferior survival rates for patients undergoing extracorporeal cardiopulmonary resuscitation [24,25]. A similar result was found in our study. Absence of ROSB represents irreversible myocardial dysfunction caused by severe malperfusion induced by either prolonged shock or interruption of the coronary blood supply. Furthermore, subsequent aortic surgery with cardioplegic arrest and circulatory arrest may also lead to second injuries to the heart and brain, which involve difficult recovery. Therefore, because of the predicted disastrous outcomes, we suggest that surgeons should carefully consider their decisions when treating ATAAD patients without ROSB after CPR. On the contrary, after excluding six patients without ROSB, the remaining 16 patients had an in-hospital survival rate of up to 60%. Based on this finding, patients with ROSB after CPR should be managed aggressively to achieve promising survival rates. In the present study, four octogenarians (cases 5, 7, 13, 15) were included, and all of them died after surgery even though a 75% (3/4) ROSB rate was presented. As reported by Piccardo et al., the in-hospital mortality rate for octogenarians who underwent emergent surgical repair for complicated ATAAD was >85%, and a medical treatment was suggested [26]. In the present study, a similar poor outcome in regard to octogenarians was also found. Therefore, surgeons may also take careful consideration before treating octogenarians in this critical scenario.

### Cerebral performance

CPC score is one of the most widely used assessments of the functional status of cardiac arrest patients. Its score ranges from 1 (good cerebral performance) to 5 (brain death). Patients with different CPC scores at discharge have significantly different survival trajectories. As reported in previous literatures, CPC 1 and 2 generally categorized as favorable neurological outcome and 3 to 5 as unfavorable outcome [27,28]. In patients who have been successfully resuscitated after cardiac arrest, favorable CPC at hospital discharge predicts better long-term outcomes than those with unfavorable CPC [29]. In the present study, approximately 90% of patients (8/9), presented with CPC 1–2 at discharge, and all of them had favorable CPC at the 3-month follow-up after discharge. There was only one patient of late mortality; case 10 died due to new cerebral infarction complicated by pneumonia at 8 months after discharge. The rest of 8 survivors all recovered well without medical events. Therefore, we believe that the functional and neurological status in cardiac arrest survivors should be a meaningful clinical outcome of no less importance than the hospital survival rate when evaluating the effectiveness of resuscitation care.

### Limitations

This study had several limitations. First, as a retrospective and non-randomized control study with a small sample size, some potential bias might have existed that could have influenced the homogeneity of the survivor and non-survivor groups. Furthermore, the small sample size may also affect the power of statistical analyses and strength of data interpretation. Second, because this crossed cohort spanned a period more than 13 years, technology involved in CPB and myocardial protection for ATAAD surgery, ACLS protocols, and CPR management may have changed over time. Finally, despite the convincing results of the present study, an extended follow-up study with more patients should be conducted in the future to evaluate the long-term outcomes of this high-risk ATAAD subgroup.

## Conclusions

The incidence of acute type A aortic dissection with preoperative cardiopulmonary resuscitation was low. However, these patients, especially those without return of spontaneous heartbeat after cardiopulmonary resuscitation, are at tremendous risk for in-hospital mortality. As a critical population, the short-term and mid-term outcomes for patients who survived to hospital discharge are promising, including the cerebral performance after discharge and 3-year survival rate.

## Supporting information

S1 Table Preoperative characteristics and surgical information in non-matched patients of the non-CPR and CPR groups. (DOC)

S2 Table Preoperative characteristics and surgical information in propensity-matched patients of the non-CPR and CPR groups. (DOC)

S3 Table Postoperative mortality and morbidity in propensity-matched patients of the non-CPR and CPR groups. (DOC)
