# Supplementary material for: Surgical results in acute type A aortic dissection with preoperative cardiopulmonary resuscitation: Survival and neurological outcome
Source: PLoS One. 2020 Aug 24;15(8):e0237989. doi: 10.1371/journal.pone.0237989 (PMC7446916; doi:10.1371/journal.pone.0237989)
Supplement: S1 Table — (DOC) [file pone.0237989.s001.doc]

**S1 Table. Preoperative characteristics and surgical information in non-matched patients of the non-CPR and CPR groups.**

| Parameters | Overall | Non-CPR | CPR | *p* Value |
| --- | --- | --- | --- | --- |
|  | n = 656 | n = 634 | n = 22 |  |
| Clinical demographics |  |  |  |  |
| Sex (female, n, %) | 194, 29.6 | 186, 29.3 | 8, 36.4 | 0.478 |
| Age (year) | 56.0 (46.5–64.9) | 55.7 (46.3–64.6) | 63.1 (54.9–76.5) | 0.021 |
| Body mass index (kg/m^2^) | 25.0 (23.0–28.0) | 25.0 (23.0–28.0) | 25.1 (23.0–27.6) | 0.704 |
| Hypertension (n, %) | 468, 71.3 | 454, 71.6 | 14, 63.6 | 0.416 |
| Diabetes mellitus (n, %) | 44, 6.7 | 41, 6.5 | 3, 13.6 | 0.186 |
| Creatinine (mg/dL) | 1.1 (0.9–1.5) | 1.1(0.9–1.4) | 1.6 (1.0–2.0) | 0.021 |
| eGFR (mL/min/1.73 m^2^) | 67.2 (49.8–86.6) | 67.3 (50.4–87.3) | 43.9 (34.8–72.2) | 0.006 |
| Preoperative condition and CPR-related profiles |  |  |  |  |
| SBP (mmHg) | 97.0 (90.0–105.0) | 98.0 (90.0–105.0) | 50.0 (45.0–57.0) | <0.001 |
| SBP <60 mmHg (n, %) | 32, 4.9 | 14, 2.2 | 18, 81.8 | <0.001 |
| Time from ED to OR (hr) | 5.1(3.8–6.4) | 5.1 (3.8–6.4) | 4.3 (2.5–6.5) | 0.183 |
| Surgical resuscitation procedures (n, %) | 47, 7.2 | 31, 4.9 | 16, 72.7 | <0.001 |
| CPR at ED (n, %) | — | — | 6, 27.3 | N/A |
| CPR duration (min) | — | — | 20.0 (5.5–30.0) | N/A |
| ROSB (n, %) | — | — | 16, 72.7 | N/A |
| Clinical presentation |  |  |  |  |
| Intractable pain (n, %) | 484, 73.8 | 471, 74.3 | 13, 59.1 | 0.111 |
| Aortic regurgitation > moderate (n, %) | 98, 14.9 | 91, 14.4 | 7, 31.8 | 0.024 |
| Hemopericardium (n, %) | 215, 32.8 | 198, 31.2 | 17, 77.3 | <0.001 |
| Cardiac tamponade (n, %) | 79, 12.0 | 63, 9.9 | 16, 72.7 | <0.001 |
| Acute myocardial infarction (n, %) | 12, 1.8 | 9, 1.4 | 3, 13.6 | <0.001 |
| Malperfusion (n, %) | 104, 15.9 | 94, 14.8 | 10, 45.5 | <0.001 |
| DeBakey type II (n, %) | 70, 10.7 | 63, 9.9 | 7, 31.8 | 0.001 |
| Femoral arterial cannulation (n, %) | 624, 95.1 | 603, 95.1 | 21, 95.5 | 0.941 |
| Axillary arterial cannulation (n, %) | 553, 84.3 | 545, 86.0 | 8, 36.4 | <0.001 |
| Aortic repair procedures |  |  |  |  |
| Entry tear exclusion (n, %) | 475, 72.4 | 458, 72.2 | 17, 77.3 | 0.604 |
| Root replacement (n, %) | 74, 11.3 | 72, 11.4 | 2, 9.1 | 0.741 |
| Isolated AsAo replacement (n, %) | 406, 61.9 | 387, 61.0 | 19, 86.4 | 0.016 |
| Arch replacement (n, %) | 186, 28.4 | 185, 29.2 | 1, 4.5 | 0.012 |
| Partial arch (n, %) | 117, 17.8 | 116, 18.3 | 1, 4.5 | 0.098 |
| Total arch (n, %) | 70, 10.7 | 70, 11.0 | 0 | 0.099 |
| Cardiopulmonary bypass time (min) | 246.0 (206.3–286.0) | 245.5 (206.0–286.0) | 264.0(206.5–359.8) | 0.163 |
| Aortic clamping time (min) | 159.5 (133.0–191.8) | 160.0 (132.8–190.3) | 155.0(130.0–209.5) | 0.895 |
| Circulatory arrest time (min) | 45.0 (35.0–63.0) | 45.0 (34.0–64.0) | 44.5 (36.8–58.5) | 0.777 |
| ACP (n, %) | 564, 86.0 | 556, 87.7 | 8, 36.4 | <0.001 |
| RCP (n, %) | 92, 14.0 | 78, 12.3 | 14, 63.6 | <0.001 |
| Hypothermia temperature (°C) | 20.0 (18.0–21.0) | 20.0 (18.0–22.0) | 20.0 (18.0–20.0) | 0.115 |
| Delayed sternum closure (n, %) | 97, 14.8 | 92, 14.5 | 5, 22.7 | 0.286 |
| ECMO support (n, %) | 19, 2.9 | 16, 2.5 | 3, 13.6 | 0.002 |
| ACP, antegrade cerebral perfusion; AsAo, ascending aorta; CPR, cardiopulmonary resuscitation; ECMO, extracorporeal membrane oxygenation; eGFR, estimated glomerular filtration rate; ED, emergency department; OR, operating room; RCP, retrograde cerebral perfusion; ROSB, return of spontaneous heartbeat; SBP, systolic blood pressure. | | | | |
