# Supplementary material for: Surgical results in acute type A aortic dissection with preoperative cardiopulmonary resuscitation: Survival and neurological outcome
Source: PLoS One. 2020 Aug 24;15(8):e0237989. doi: 10.1371/journal.pone.0237989 (PMC7446916; doi:10.1371/journal.pone.0237989)
Supplement: S2 Table — (DOC) [file pone.0237989.s002.doc]

**S2 Table. Preoperative characteristics and surgical information in propensity-matched patients of the non-CPR and CPR groups.**

| Parameters | Overall | Non-CPR | CPR | *p* Value |
| --- | --- | --- | --- | --- |
|  | n = 44 | n = 22 | n = 22 |  |
| Clinical demographics |  |  |  |  |
| Sex (female, n, %) | 16, 36.4 | 8, 36.4 | 8, 36.4 | 0.999 |
| Age (year) | 62.3 (53.0–76.5) | 60.6 (51.8–77.3) | 63.1 (54.9–76.5) | 0.647 |
| Body mass index (kg/m^2^) | 25.5 (23.2–27.9) | 25.6 (23.9–28.3) | 25.1 (23.0–27.6) | 0.549 |
| Hypertension (n, %) | 33, 75.0 | 19, 86.4 | 14, 63.6 | 0.082 |
| Diabetes mellitus (n, %) | 3, 6.8 | 0 | 3, 13.6 | 0.073 |
| Creatinine (mg/dL) | 1.3 (0.9–1.7) | 1.1 (0.8–1.4) | 1.6 (1.0–2.0) | 0.087 |
| eGFR (mL/min/1.73 m^2^) | 57.8 (39.3–79.7) | 61.0 (50.7–83.7) | 43.9 (34.8–72.2) | 0.082 |
| Preoperative condition and CPR-related profiles |  |  |  |  |
| SBP (mmHg) | 55.0 (50.0–62.0) | 58.5 (55.0–66.3) | 50.0 (45.0–57.0) | 0.001 |
| SBP <60 mmHg (n, %) | 30, 68.2 | 12, 54.5 | 18, 81.8 | 0.052 |
| Time from ED to OR (hr) | 4.4 (2.6–6.2) | 4.4 (2.8–6.1) | 4.3 (2.5–6.5) | 0.878 |
| Surgical resuscitation procedures (n, %) | 29, 65.9 | 13, 59.1 | 16, 72.7 | 0.340 |
| CPR at ED (n, %) | — | — | 6, 27.3 | N/A |
| CPR duration (min) | — | — | 20.0 (5.5–30.0) | N/A |
| ROSB (n, %) | — | — | 16, 72.7 | N/A |
| Clinical presentation |  |  |  |  |
| Intractable pain (n, %) | 27, 61.4 | 14, 63.6 | 13, 59.1 | 0.757 |
| Aortic regurgitation > moderate (n, %) | 11, 25.0 | 4, 18.2 | 7, 31.8 | 0.296 |
| Hemopericardium (n, %) | 35, 79.5 | 18, 81.8 | 17, 77.3 | 0.709 |
| Cardiac tamponade (n, %) | 34, 77.3 | 18, 81.8 | 16, 72.7 | 0.472 |
| Acute myocardial infarction (n, %) | 4, 9.1 | 1, 4.5 | 3, 13.6 | 0.294 |
| Malperfusion (n, %) | 16, 36.4 | 6, 27.3 | 10, 45.5 | 0.210 |
| DeBakey type II (n, %) | 11, 25.0 | 4, 18.2 | 7, 31.8 | 0.296 |
| Femoral arterial cannulation (n, %) | 43, 97.7 | 22, 100 | 21, 95.5 | 0.312 |
| Axillary arterial cannulation (n, %) | 24, 54.5 | 16, 72.7 | 8, 36.4 | 0.015 |
| Aortic repair procedures |  |  |  |  |
| Entry tear exclusion (n, %) | 34, 77.3 | 17, 77.3 | 17, 77.3 | 0.999 |
| Root replacement (n, %) | 4, 9.1 | 2, 9.1 | 2, 9.1 | 0.999 |
| Isolated AsAo replacement (n, %) | 36, 81.8 | 17, 77.3 | 19, 86.4 | 0.434 |
| Arch replacement (n, %) | 5, 11.4 | 4, 18.2 | 1, 4.5 | 0.154 |
| Partial arch (n, %) | 4, 9.1 | 3, 13.6 | 1, 4.5 | 0.294 |
| Total arch (n, %) | 1, 2.3 | 1, 4.5 | 0 | 0.312 |
| Cardiopulmonary bypass time (min) | 251.0 (182.5–350.3) | 229.0 (177.3–312.3) | 264.0 (206.5–359.8) | 0.250 |
| Aortic clamping time (min) | 155.0 (130.8–203.5) | 154.5 (129.3–178.5) | 155.0 (130.0–209.5) | 0.778 |
| Circulatory arrest time (min) | 44.5 (36.0–56.8) | 46.0 (36.0–57.8) | 44.5 (36.8–58.5) | 0.944 |
| ACP (n, %) | 24, 54.5 | 16, 72.7 | 8, 36.4 | 0.015 |
| RCP (n, %) | 20, 45.5 | 6, 27.3 | 14, 63.6 | 0.015 |
| Hypothermia temperature (°C) | 20.0 (18.0–20.0) | 20.0 (18.0–20.5) | 20.0 (18.0–20.0) | 0.454 |
| Delayed sternum closure (n, %) | 11, 25.0 | 6, 27.3 | 5, 22.7 | 0.728 |
| ECMO support (n, %) | 4, 9.1 | 1, 4.5 | 3, 13.6 | 0.294 |
| ACP, antegrade cerebral perfusion; AsAo, ascending aorta; CPR, cardiopulmonary resuscitation; ECMO, extracorporeal membrane oxygenation; eGFR, estimated glomerular filtration rate; ED, emergency department; OR, operating room; RCP, retrograde cerebral perfusion; ROSB, return of spontaneous heartbeat; SBP, systolic blood pressure. | | | | |
