# Supplementary material for: Surgical results in acute type A aortic dissection with preoperative cardiopulmonary resuscitation: Survival and neurological outcome
Source: PLoS One. 2020 Aug 24;15(8):e0237989. doi: 10.1371/journal.pone.0237989 (PMC7446916; doi:10.1371/journal.pone.0237989)
Supplement: S3 Table — (DOC) [file pone.0237989.s003.doc]

**S3 Table. Postoperative mortality and morbidity in propensity-matched patients of the non-CPR and CPR groups.**

| Parameters | Overall | Non-CPR | CPR | *p* Value |
| --- | --- | --- | --- | --- |
|  | n = 44 | n = 22 | n = 22 |  |
| In-hospital mortality (n, %) | 17, 38.6 | 4, 18.2 | 13, 59.1 | 0.005 |
| Bleeding (n, %) | 4, 9.1 | 1, 4.5 | 3, 13.6 | 0.294 |
| Myocardial failure (n, %) | 7, 15.9 | 1, 4.5 | 6, 27.3 | 0.039 |
| Brain stem failure (n, %) | 4, 9.1 | 2, 9.1 | 2, 9.1 | 0.999 |
| Sepsis (n, %) | 2, 4.5 | 0 | 2, 9.1 | 0.148 |
| Renal failure (n, %) | 4, 9.1 | 1, 4.5 | 3, 13.6 | 0.294 |
| Transfusion at 24 hr after surgery |  |  |  |  |
| RBC (units) | 6.0 (6.0–13.5) | 6.0 (6.0–15.0) | 6.0 (5.5–12.5) | 0.561 |
| Plasma (units) | 6.0 (4.0–12.0) | 6.0 (4.0–13.0) | 6.0 (4.0–10.5) | 0.748 |
| Platelet (units) | 12.0 (12.0–24.0) | 18.0 (12.0–27.0) | 12.0 (12.0–24.0) | 0.384 |
| Re-exploration for bleeding (n, %) | 9, 20.5 | 4, 18.2 | 5, 22.7 | 0.709 |
| Atrial fibrillation (n, %) | 3, 6.8 | 2, 9.1 | 1, 4.5 | 0.550 |
| Brain stroke (n, %) | 10, 22.7 | 4, 18.2 | 6, 27.3 | 0.472 |
| Infarction (n, %) | 9, 20.5 | 3, 13.6 | 6, 27.3 | 0.262 |
| Hemorrhage (n, %) | 3, 6.8 | 2, 9.1 | 1, 4.5 | 0.550 |
| Delirium (n, %) | 10, 22.7 | 5, 22.7 | 5, 22.7 | 0.999 |
| Seizure (n, %) | 5, 11.4 | 3, 13.6 | 2, 9.1 | 0.635 |
| Visceral ischemia (n, %) | 2, 4.5 | 2, 9.1 | 0 | 0.148 |
| Limb ischemia (n, %) | 2, 4.5 | 1, 4.5 | 1, 4.5 | 0.999 |
| Malperfusion-related complications (n, %) | 15, 34.1 | 8, 36.4 | 7, 31.8 | 0.750 |
| Pneumonia (n, %) | 9, 20.5 | 4, 18.2 | 5, 22.7 | 0.709 |
| ICU stay (days) | 5.0 (2.3–8.8) | 5.5 (3.0–8.0) | 3.5 (0.5–17.0) | 0.688 |
| Hospital stay (days) | 16.5 (4.3–26.8) | 17.0 (9.8–21.3) | 13.5 (0.5–30.3) | 0.411 |
| ICU, intensive care unit. | | | | |
